# Application of Modified Sliding Anastomosis in the Repair of Aortic Coarctation

**DOI:** 10.1155/2020/3805385

**Published:** 2020-05-14

**Authors:** Wangping Chen, Chengming Fan, Shiyuan Tang, Wenwu Zhou, Chukwuemeka Daniel Iroegbu, Jiarong Li, Xiaoming Wu, Jinfu Yang

**Affiliations:** ^1^Department of Cardiovascular Surgery, The Second Xiangya Hospital of Central South University, Middle Renmin Road 139, Changsha 410000, China; ^2^Department of Cardiovascular Surgery, The People's Hospital of Hunan Province, West Jiefang Road 61, Changsha 410000, China

## Abstract

**Objectives:**

To evaluate the early and midterm results of a modified sliding anastomosis technique in patients with aortic coarctation.

**Materials and Methods:**

In this study, we reported a new repair method and compared the early and midterm outcome(s) with a conventional surgical approach for the management of patients with aortic coarctation. Forty-eight aortic coarctation patients with a narrowed segment length longer than 2 cm were operated at our department's pediatric surgical division. Excision of the coarctation and end-to-end anastomosis was carried out in twenty-five patients (control group). In contrast, a modified sliding technique was used for twenty-three cases in the observation group. Other accompanying cardiac anomalies simultaneously repaired included ventricular septal defect and patent ductus arteriosus. All patients received 1.5-10 years of postoperative echocardiographic follow-up.

**Results:**

This is a retrospective study carried out between January 2005 and June 2018. The study population consisted of forty-eight patients, which included twenty-six male and twenty-two female patients, with an average age of 5.2 ± 1.9 months (range, 28 days to 1 year). There was no mortality. The operative time, the number of intercostal artery disconnection, the drainage volume, and arm-leg systolic pressure gradient postoperation were less in the observation group as compared to the control group (*p* < 0.05). Also, cases with an anastomotic pressure gradient exceeding 10 mmHg during follow-up were less in the observation group as compared to the control group (*p* < 0.05). The postoperative complications encountered were chylothorax (control group 2 cases vs. observation group 0) and pulmonary atelectasis (control group 4 cases vs. observation group 1). They all, however, recovered after conservative treatment. Three patients in the control group underwent balloon angioplasty (reintervention) postoperative 2-4 years due to an increase in the anastomotic pressure gradient (>20 mmHg). After reintervention, the anastomotic pressure gradient reduced to 14 mmHg, 15 mmHg, and 17 mmHg, respectively.

**Conclusions:**

For long segment aortic coarctation patients (longer than 2 cm), the use of the modified sliding anastomotic technique effectively helps to retain more autologous tissues, enlarge the diameter of the anastomosis, and decrease anastomotic tension and vascular injury. Therefore, this technique provides a new idea for the surgical treatment of aortic coarctations.

## 1. Introduction

Aortic coarctation, first discovered by Morgagni in 1760 [1], is a common congenital heart defect that causes a limited or complete stoppage of blood flow due to its narrow lumen. It accounts for about 7% of all congenital heart diseases with a reported 1.5 : 1 male to female predominance ratio [2]. Although there are several methods or surgical techniques used in treating aortic coarctation, Crafoord in 1947 was the first to describe the surgical procedure for narrow segment resection and end-to-end anastomosis [3]. In 1960, Vossschulte and colleagues used a patch angioplasty technique to correct aortic coarctation, successfully relieving the aortic pressure gradient [4]. Left subclavian valvuloplasty, first used in the 60s and widely used in the 80s, now is often used for the treatment of aortic coarctation in neonates and infants [5, 6].

In 1975, Edie and colleagues succeeded in applying artificial vascular bypass grafting to cure patients with an aortic constriction that was difficult to dissect and expose [7]. Besides, minimally invasive procedures such as balloon angioplasty and endovascular stents are also treatment options for aortic coarctation [8, 9]. However, these methods have their shortcomings, such as excessive anastomotic tension, the high incidence rate of re-narrowing, postoperative aneurysm formation, and the absence of growth potential for artificial blood vessels, which lead to a poor postoperative outcome [10–13]. Hence, the need and/or thought of reintervention(s) poses a wearing burden on cardiovascular surgeons. Therefore, with that in mind, an ideal surgical procedure for aortic coarctation surgery will be a long-cherished dream for cardiovascular surgeons.

## 2. Materials and Methods

### 2.1. Patients

**Figure 1:**
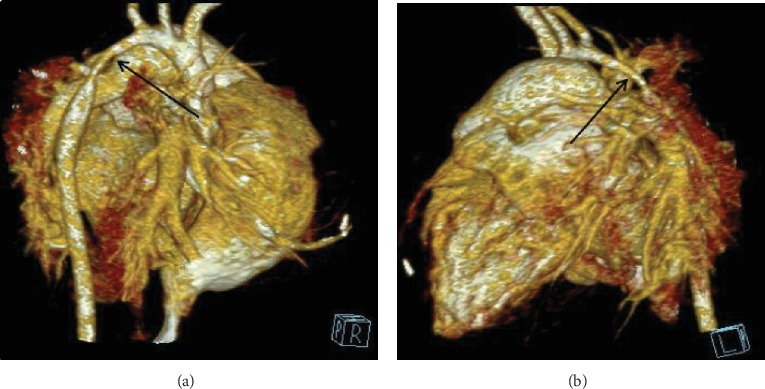
Classic CTA images: arrows highlight the aortic coarctation (COA).

Prior approval from the Research Ethics Committee of the Second Xiangya Hospital was obtained. We obtained written, informed consent from all participants. Methods used and applied to the research were carried out under relevant guidelines and regulations. All patients included in this study met the following criteria: (i) narrowed aortic segment length longer than 2 cm and (ii) isolated aortic coarctation or only combined with ventricular septal defect and/or patent ductus arteriosus. Our study excluded the following: (i) patients with narrowed aortic segment length shorter than 2 cm or (ii) patients with aortic coarctation combined with complex cardiac anomalies such as aortic arch dysplasia and transposition of great arteries. From January 2005 to June 2018, forty-eight aortic coarctation patients with narrowed segment length, longer than 2 cm, were surgically operated in our institution (Table 1). All patients had lower extremity arterial pulsation weakening, and their upper extremity blood pressure was higher than that of the lower extremity. Systolic murmurs of different degrees could be heard in the subscapular area. Patients' diagnoses were all ascertained through computer tomographic angiography (Figure 1).

### 2.2. Surgical Technique

The fourth intercostal incision of the left posterior lateral chest was applied for patients of aortic coarctation with or without patent ductus arteriosus. The median sternal incision was applied for patients of aortic coarctation with ventricular septal defect, and the concomitant ventricular septal defect was repaired under cardiopulmonary bypass with hypothermia. The blood flow in both sides of the carotid was maintained normally at the whole duration of the procedure, and therefore, the cerebral perfusion was not decreased significantly.

#### 2.2.1. Coarctation Resection and End-to-End Anastomosis

**Figure 2:**
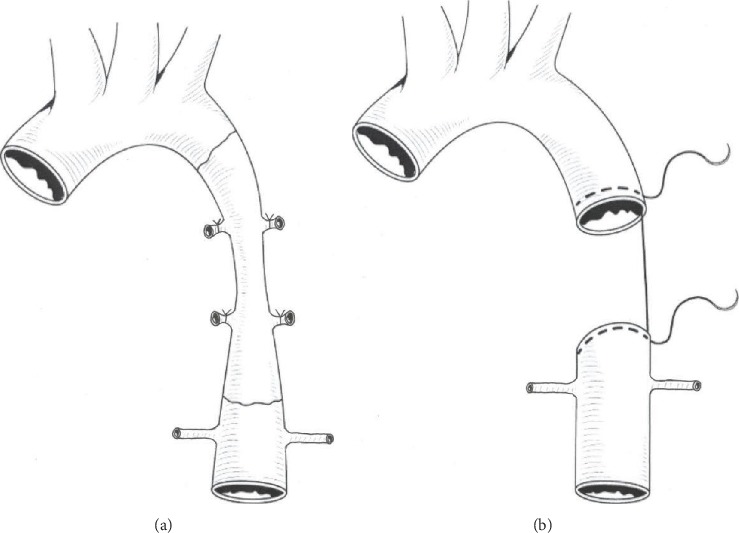
Resection and end-to-end anastomosis.

In this group (control group), the narrowed aortic segment was excised entirely and an end-to-end anastomosis was performed. After thorough exposure of the aorta, we used the vascular clamp to block the narrowed part of the aorta at both distal and proximal ends. Then, we resected the narrowed segment of the aorta and performed an end-to-end continuous anastomosis using two 6-0 prolene sutures, as shown in Figure 2. No interposition graft material was used. The aortic clamp time was 18.6 ± 2.1 mins.

#### 2.2.2. Modified Sliding Anastomosis Technique

**Figure 3:**
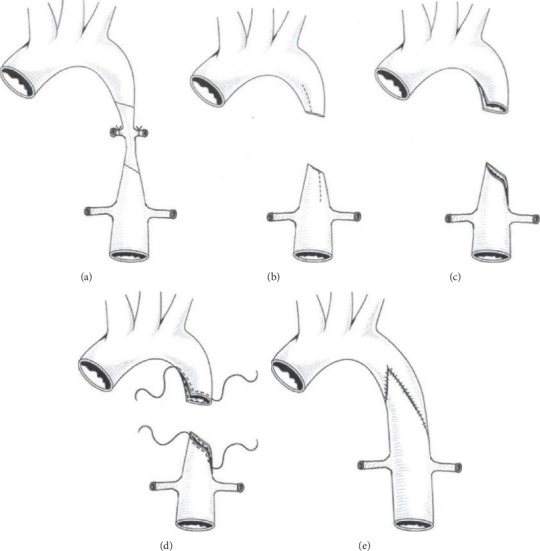
Partial resection of the stenotic part and modified sliding anastomosis.

For this group (observation group), after entire exposure of the aorta, we partly removed its stenotic segment. We also preserved some stenosed vessels at the distal and proximal ends of the aorta (Figure 3(a)). The proximal vessels were incised longitudinally at the small curved side to the normal width, and the distal vessels were also longitudinally incised at the large curved side in equal length (Figures 3(b) and 3(c)). After adequately trimming the proximal and distal aortic vessels, both segments were pulled and slid towards each other. The entire end of the vessel wall was inserted into the other end to suture the incisions correspondingly. Continuous suture technique with two 6-0 prolene sutures was applied (Figures 3(d) and 3(e)). No interposition graft material was inserted. The aortic clamp time was 19.5 ± 2.4 mins.

### 2.3. Statistical Analysis

Data are expressed as the mean ± SE. All calculations were performed using the Statistical Product and Service Solutions 20 software (SPSS Institute). *t*-test, *χ*^2^ test, log-rank test, and Gehan-Breslow-Wilcoxon test were performed to determine differences between the two groups. The critical alpha level for these analyses was set at *p* < 0.05.

## 3. Results

There were twenty-six males and twenty-two females, with an average age of 5.2 ± 1.9 months (range, 28 days to 1 year), and an average body weight of 5.9 ± 1.3 kg (range 3.5 kg to 12 kg). Among them, ten cases were isolated aortic coarctation (COA), eighteen cases were combined with PDA, and twelve cases were combined with VSD, while eight cases were combined with both PDA and VSD. The aortic clamp time was similar in both groups (control group 18.6 ± 2.1 mins vs. observation group 19.5 ± 2.4 mins, *p* = 0.208). The operative time, the number of intercostal artery disconnection, the drainage volume, and arm-leg systolic pressure gradient postoperation were decreased in the observation group as compared to the control group (Table 2, *p* < 0.05). The common complications encountered were chylothorax (control group 2 cases vs. observation group 0) and pulmonary atelectasis (control group 4 cases vs. observation group 1). Shown in Table 3 were the surgical repair details for the accompanying congenital cardiac abnormalities. There were no recorded mortalities.

**Figure 4:**
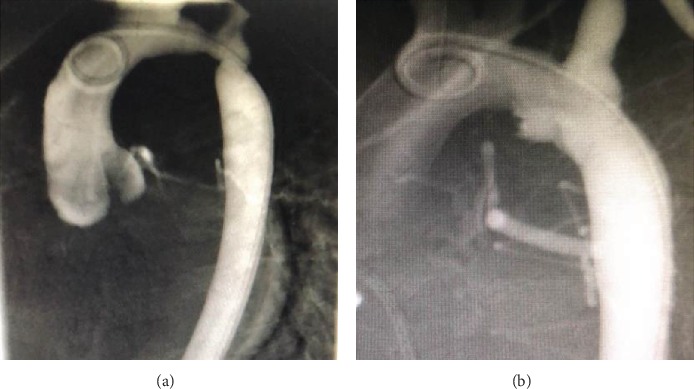
Representative illustrations before (a) and after (b) balloon angioplasty for recoarctation.

**Figure 5:**
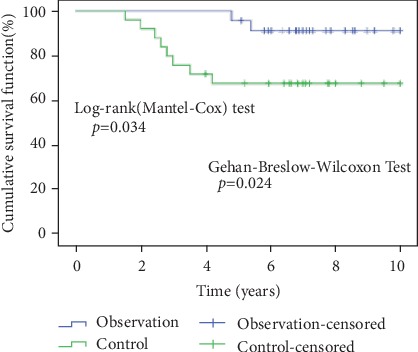
Kaplan-Meier curve showed that there was a significant difference between the two groups in the number of patients whose anastomotic pressure gradient exceeded 10 mmHg during follow-up.

Patients were all followed up echocardiographically for 1.5-10 years. In the control group, eight patients had an anastomotic pressure gradient of >10 mmHg, while three had an anastomotic pressure gradient of 20-50 mmHg. We carried out balloon angioplasty (reintervention) in three patients (control group) 2-4 years after the initial procedure due to an increase in the anastomotic pressure gradient (>20 mmHg). Under general anesthesia and using a contrast agent-inflated balloon, the angioplasty for 3 cases of the restenotic aorta was performed with a retrograde arterial route. The initial diameter of the balloon was slightly less than or equal to the diameter of the descending aorta at the level of the diaphragm, and the length of the balloon was 20 mm. Therefore, the contrast agent-inflated balloon with a size of 12 mm∗20 mm was used in two cases and 10 mm∗20 mm in one case. The balloon was positioned across the recoarctation; then, the balloon was inflated to five atmospheres of pressure or higher, according to the manufacturer's recommendations. After a total of two to five times of inflation, the balloon catheter was removed; then the aortography and measurement of systolic gradient pressure across the recoarctation were repeated (Figure 4). After dilatation, the systolic pressure gradient of these three patients reduced to 14 mmHg, 15 mmHg, and 17 mmHg, respectively. Apart from the three cases mentioned above, there were no significant changes during the follow-up period in the control group. In the observation group, two patients had an echocardiographic anastomotic pressure gradient measurement of 10 mmHg and 13 mmHg, respectively. There were no reintervention(s) for the observation group. Also, there were no significant changes recorded or observed during the follow-up period. Statistical analysis showed significant differences in the number of patients with anastomotic pressure gradient exceeded 10 mmHg in both groups (Figure 5).

## 4. Discussion

Aortic coarctation accounts for 5% to 8% of all congenital heart diseases, with an incidence rate of three to four cases per 10 000 live births [14–16]. Classically, the coarctation is located at the thoracic aorta distal to the origin of the left subclavian artery [2]. Aortic coarctation is clinically divided into a preductal and a postductal types according to the coarctation site and the arterial duct or ligament. Most patients are diagnosed with aortic coarctation based on their symptoms and auxiliary examinations. As reported in the literature [17], patients with aortic lumen diameter < 50% of the normal lumen at the coarctation site and/or a pressure gradient > 20 mmHg at rest should undergo surgery [17].

Traditional surgical methods for aortic coarctation include coarctation resection plus end-to-end anastomosis, patch enlargement plasty, left subclavian valvuloplasty, and artificial vascular bypass grafting. However, these surgical methods have their disadvantages, such as excessive anastomotic tension, high renarrowing rate, postoperative aneurysm formation, and the lack of growth potential, especially for patients with longer stenotic segments. Furthermore, dissociating longer descending aorta and ligating intercostal arteries only cause more damage. With regard to balloon angioplasty, its indication is in a narrow range and usually applied for mild aortic coarctation. Therefore, to alleviate these medical setbacks, we used a new anastomotic technique for the treatment of twenty-three patients with aortic coarctation longer than 2 cm with satisfactory clinical results. Inspired by the sliding technique used for tracheal stenosis, we employed a slant-wise resection and sliding anastomotic technique for the partially stenosed aortic lesion; we hence termed modified sliding anastomosis technique.

For patients with long stenotic segments, coarctation resection and an end-to-end anastomosis would require extensive dissection of the distal and proximal ends of the aorta. These will, however, in return, lead to restenosis [18] and high anastomotic tension due to the remarkable distance between both ends. As for blood vessel patch angioplasty and artificial bypass grafting methods, the use of artificial or biological material is ideal. A large number of patients with aortic coarctation are mostly children, and as the child grows, the aortic lumen becomes more extensive, leading to a mismatch between the autologous blood vessel and the implant material. This extensive stretching of the lumen will, however, eventually result in postoperative restenosis and aneurysm formation, thereby, affecting long-term prognosis [19]. To avoid the setbacks encountered with the traditional techniques, we adopted the slide tracheoplasty and considered the use of sliding anastomosis in the treatment of patients with aortic coarctation. The specific surgical steps taken are as follows: (i) with a slantwise approach, partially resect the stenotic aortic vessels; (ii) longitudinally incise the proximal vessels at the small curved side to its width and then incise the distal vessels at the large curved side in equal length; (iii) after adequately trimming the distal and proximal aortic vessels, pull and slide the two segments of the aorta towards each other and insert the entire end of the vessel wall into the other end of the incision to suture correspondingly. This technique enlarges the lumen, reduces the length of the aortic resection, and increases the lumen area. Comparing the standard aortic coarctation procedure and modified sliding anastomotic procedure, classic end-to-end anastomosis completely resects stenotic segments, implores extensive resections, and causes more significant aortic injuries. However, with the sliding anastomosis technique, only a small portion of the stenotic aorta is being cut and then longitudinally incising the aortic wall to its width. Finally, the advantages of the modified sliding technique include (i) few intercostal artery disconnections and minimal aortic tissue dissections, (ii) low anastomotic tension which effectively prevents postoperative restenosis [20], (iii) the intact vascular endothelium which prevents postoperative infection and aneurysm formation [21], and (iv) potential growth capacity of the reserved autologous vascular tissue. This technique is expected to be the preferred surgical procedure for patients with long-segment aortic coarctation.

## 5. Limitations

We acknowledge the fact that our study has some limitations. As a single-center study, the research involved a small sample size of forty-eight patients with a relatively short follow-up period. A prospective multicenter study with a comparatively bigger sample size will be essential to confirm the effect of this surgical method.

## 6. Conclusion

The modified sliding technique has the advantage of retaining more autologous tissues, enlarging the lumen of the anastomose aorta, preventing anastomotic stoma tensions, and limiting surgical injuries. It also provides a new method for the surgical treatment of aortic coarctation, especially in patients with a long stenosed aortic segment.

## Figures and Tables

**Table 1:** Preoperative data from the two groups.

	Observation group (*n* = 23)	Control group (*n* = 25)	*p* value
Gender (male/female)	12/11	14/11	0.666
Age (month)	5.0 ± 1.5	5.4 ± 2.1	0.417
Body weight (kg)	5.7 ± 0.9	6.0 ± 1.5	0.285
Arm-leg systolic gradient (mmHg)	55 ± 16	53 ± 15	0.673
Preductal type COA	15	18	
Postductal type COA	8	7	

**Table 2:** Postoperative data of two groups.

	Observation group (*n* = 23)	Control group (*n* = 25)	*p* value
Operative time (min)	71.9 ± 11.3	80.1 ± 13.2	0.025
Intercostal artery disconnection (pairs)	1.5 ± 0.5	2.5 ± 0.6	<0.001
Aortic clamp time (min)	19.5 ± 2.4	18.6 ± 2.1	0.208
Drainage volume (ml)	37.1 ± 16.3	50.6 ± 21.2	0.018
Arm-leg systolic gradient (mmHg)	3 ± 3	6 ± 5	0.020
Chylothorax (case)	0	2	
Pulmonary atelectasis (case)	1	4	
Anastomosis pressure difference 10-20 mmHg (case)	2	5	
Anastomosis pressure difference 20-50 mmHg (case)	0	3	
Anastomosis pressure difference higher than 10 mmHg (case)	2	8	0.034

**Table 3:** Concomitant operations during COA correction.

Operation	Patients
PDA ligation or suture	18
VSD closure	12
PDA ligation or suture+VAD closure	8

## Data Availability

The data used to support the findings of this study are available from the corresponding author upon request.
